# Supplementary material for: Photosynthetic, Respirational, and Growth Responses of Six Benthic Diatoms from the Antarctic Peninsula as Functions of Salinity and Temperature Variations
Source: Genes (Basel). 2022 Jul 16;13(7):1264. doi: 10.3390/genes13071264 (PMC9324188; doi:10.3390/genes13071264)
Supplement: Supplementary file 1 [file genes-13-01264-s001.zip › genes-1814388-SI.pdf]

Table S1. Confidence intervals for salinity dependent growth rates and temperature-dependent growth rates, photosynthetic rates and respirational rates in the studied Antarctic benthic diatoms isolated from Carlini Station, King George Island Potter Cove in austral summer 2020 (January/February).

	Confidence intervals	
**Species**	**2.5%**	**97.5%**
Navicula criophiliforma		
maximal photosynthetic rate	231.663	353.517
optimum photosynthetic temperature	5.934	16.303
maximum photosynthetic temperature	30.825	35.868
maximum respirational rate	-212.388	-159.586
optimum respirational temperature	28.002	-81.961
maximum respirational temperature	40.141	43.668
Chamaepinnularia gerlachei		
maximum growth rate (salinity)	0.537	0.621
optimum growth salinity	2.593	10.457
maximum growth salinity	87.282	100.156
maximal growth rate	0.229	0.659
optimum growth temperature	4.620	21.302
maximum growth temperature	23.218	34.486
maximal photosynthetic rate	67.586	115.146
optimum photosynthetic temperature	6.301	17.866
maximum photosynthetic temperature	29.657	35.282
maximum respirational rate	-113.587	-81.961
optimum respirational temperature	28.906	32.279
maximum respirational temperature	39.726	42.616
Navicula concordia		
maximal photosynthetic rate	43.144	54.717
optimum photosynthetic temperature	13.054	18.272
maximum photosynthetic temperature	34.235	37.026
maximum respirational rate	-31.895	-26.055
optimum respirational temperature	26.108	9.183
maximum respirational temperature	41.851	47.030
Nitzschia annewillemsiana		
maximal photosynthetic rate	34.524	52.401
optimum photosynthetic temperature	7.674	17.282
maximum photosynthetic temperature	31.121	35.854
maximum respirational rate	-53.619	-37.302
optimum respirational temperature	23.856	29.366
maximum respirational temperature	39.359	46.399
***Planothidium* sp.**		
maximal photosynthetic rate	37.561	69.429
optimum photosynthetic temperature	4.275	18.757
maximum photosynthetic temperature	29.766	36.829
maximum respirational rate	-102.387	-65.730
optimum respirational temperature	24.990	31.081

maximum respirational temperature	38.950	46.994
Psammothidium papilio		
maximum growth rate (salinity)	0.376	0.455
optimum growth salinity	3.400	7.167
maximum growth salinity	26.997	31.529
Maximal growth rate (temperature)	0.184	0.406
optimum growth temperature	-4.373	17.325
Maximum growth temperature	21.883	34.038
maximal photosynthetic rate	28.695	142.941
optimum photosynthetic temperature	-6.319	12.304
maximum photosynthetic temperature	21.837	30.219
maximum respirational rate	-123.938	-76.441
optimum respirational temperature	25.562	31.759
maximum respirational temperature	38.754	46.231
